# Impact of a Clinical Decision Support System on the Efficiency and Effectiveness of Performing Medication Reviews in Community Pharmacies: A Randomized Controlled Trial

**DOI:** 10.3390/healthcare12232491

**Published:** 2024-12-09

**Authors:** Armin Dabidian, Florian Kinny, Melina Steichert, Sabina Schlottau, Anke Bartel, Holger Schwender, Stephanie Laeer

**Affiliations:** 1 Institute of Clinical Pharmacy and Pharmacotherapy, Heinrich Heine University Düsseldorf, Universitaetsstrasse 1, 40225 Duesseldorf, Germany; florian.kinny@hhu.de (F.K.); melina.steichert@hhu.de (M.S.); sabina.schlottau@hhu.de (S.S.); anke.bartel@hhu.de (A.B.); stephanie.laeer@hhu.de (S.L.); 2 Mathematical Institute, Heinrich Heine University Düsseldorf, Universitaetsstrasse 1, 40225 Duesseldorf, Germany; holger.schwender@hhu.de

**Keywords:** clinical decision support system (CDSS), medication review (MR), community pharmacy, pharmacists, medication safety, medication therapy management, eHealth

## Abstract

Background: Clinical decision support systems (CDSSs) already support community pharmacists in conducting medication reviews (MRs) by identifying important information on interactions and suggesting clinical solutions. However, their impact in terms of quality and time savings is widely unexplored. The aim of our study was to investigate whether MRs are performed faster and better with or without using a CDSS. Methods: In a randomized controlled study with a cross-over design, 71 pharmacists performed a total of four MRs, two with and two without the use of a CDSS. The primary endpoint was defined as the time required for the MRs. The secondary endpoints were the number of predefined relevant drug-related problems (DRPs) detected and pharmacist satisfaction. Results: Without the use of a CDSS, pharmacists needed between 25.7% and 30.7% more time to perform a MR than with a CDSS. In addition, significantly more relevant DRPs were detected in the MRs with CDSS than without CDSS (70% vs. 50%; *p* = 0.0037). Furthermore, participants stated that they felt more confident using a CDSS for MRs than without. Conclusions: Our results demonstrate that MRs can be performed both faster and better when using a CDSS than without. Consequently, community pharmacists benefit from the use of CDSSs for MRs, as do patients in terms of their drug therapy safety.

## 1. Introduction

Germany has one of the oldest populations in the world, with a median age of 45.1 years and 22% aged 65 or older in 2022 [1,2]. The prevalence of multimorbidity increases with age, as does the number of medications taken [3,4]. Although there is no standardized definition of the term polypharmacy, it is often used when a patient has to take five or more medications [3,5]. In Germany, the prevalence of polypharmacy has been reported, with a wide range from 26.7% to 47.1% [6,7,8]. Polypharmacy is associated with health-related problems such as adverse drug events (ADEs), hospitalization, increased mortality, and other drug-related problems (DRPs) [3,9]. A DRP is defined as “an event or circumstance involving drug therapy that actually or potentially interferes with desired health outcomes” [10]. However, polypharmacy is often necessary for multimorbid patients.

One effective method of monitoring and improving patients’ medication is through a medication review (MR) by community pharmacists. According to the Pharmaceutical Care Network Europe (PCNE), a MR is defined as “[…] a structured evaluation of a patient’s medicines with the aim of optimizing medicines use and improving health outcomes. This entails detecting drug-related problems (DRP) and recommending interventions” [11]. Previous studies have already shown that MRs performed by community pharmacists can be beneficial for various patients, e.g., for blood pressure, HbA1c levels, cardiovascular risk, ADEs, or medication adherence [12].

Since 2022, pharmacists in community pharmacies in Germany have been authorized to provide patients certain pharmaceutical services that are reimbursed by statutory health insurance (SHI) [13]. One of these services is extended medication counseling for patients with polymedication who are prescribed five or more systemically effective long-term medications or inhalants [14]. A central element of the extended medication counseling service includes MRs and the associated detection of DRPs. In addition to the MR procedure, a patient consultation is required before and after the MR to be eligible for reimbursement by the SHI. The objectives of extended medication counseling are to increase the effectiveness and safety of drug therapy, to improve adherence to treatment, and to strengthen cooperation between the healthcare professions. This is an important and welcome step in the German healthcare system in view of the demographic challenges that lie ahead. Patients are entitled to medication counseling every 12 months. In the event of significant changes, the service can be provided and reimbursed again before the 12-month period [14].

Despite the positive effects of a MR on several clinical parameters, implementation remains difficult due to various hurdles. One important practical hurdle is the time-consuming process during opening hours, where costs and availability of community pharmacists are the major limitations to this service [15,16]. Therefore, digital tools such as clinical decision support systems (CDSSs) could help pharmacists overcome these time constraints and may contribute to improving patient safety [16,17]. CDSSs are used by physicians, pharmacists, other healthcare professionals, and patients to provide information to support clinical decision making [18]. The first CDSSs with a focus on patient medication were already available in the 1960s [18,19]. These CDSSs check, for example, the correct dosage, contraindications, drug–drug interactions, or drug–disease interactions. In recent years, computerized CDSSs have become increasingly important and are also becoming more common in community pharmacies. They often follow decision tree-based models and make recommendations on the correct medication for patients [19]. Several studies have also shown that CDSSs in the pharmaceutical field can have a positive impact on various endpoints, such as drug–drug interactions or the reduction in inappropriate medication prescription in the elderly or pregnant women [20]. The first computerized CDSSs are already available in community pharmacies in Germany to support pharmacists performing MRs.

The aim of this study was to investigate whether a CDSS can save time when community pharmacists perform MRs and whether a CDSS has an impact on the quality of MRs. As CDSS, the software “MediCheck” (Version 4.3.6) was used, which is an approved medical device. Also, it has already been shown that “MediCheck” has a positive effect on the quality of MRs [21,22]. Finally, the level of acceptance of this CDSS among the participating community pharmacists for MRs was investigated.

## 2. Materials and Methods

### 2.1. Participants and Study Design

**Figure 1:**
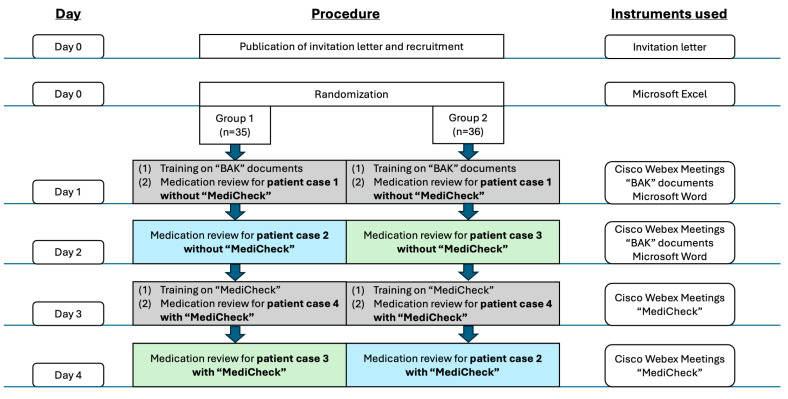
Study procedure and time schedule. BAK = “Bundesapothekerkammer” (Federal Chamber of Pharmacists).

The “Medi-fASt”study (“Medikationsanalysen für Apotheken—Softwareunterstützt und therapieoptimiert”) is a randomized, controlled cross-over study. The study was conducted entirely online. Participants were recruited via an invitation letter prepared by employees of the Institute of Clinical Pharmacy and Pharmacotherapy Duesseldorf, Germany. The invitation letter was published in the newsletter and on the social media platform of the company “pharma4u GmbH”, which provided the CDSS “MediCheck” during the study. Notably, “pharma4u GmbH” focuses on the development of digital, web-based software, apps, and content for pharmaceutical professionals and provides its services to more than 10,000 customers [23]. Furthermore, an advertisement for participation in the study was published in a German journal for pharmacists (“Pharmazeutische Zeitung”). In addition to information regarding the study procedure and requirements for participation, the invitation letter included the contact details (e-mail address and telephone number) of the study director (A.D.), in case further information was required or the individuals wished to register for the study. The study itself took place between April and May 2024, with each participant engaged in the study for a period of four days over the course of one week. Interested parties were offered the option of participating in the study for one week in either April or May. The inclusion criteria for participation in the study were as follows: (1) possession of a German license to practice as a pharmacist, (2) current employment in a community pharmacy at the time of the study, (3) no establishment of MR as a standard service in the employed pharmacy, (4) possession of a PC or laptop with “Microsoft Word” 2021 software, and (5) a signed declaration of consent to participate in the study and to data protection. The recruitment period ended one week prior to the respective study week. To protect the participants’ privacy, each participant was assigned a pseudonym for the duration of the study. Prior to the start of the study, the participants were randomly assigned to one of two groups. The study was approved by the Ethics Committee of the Medical Faculty of Heinrich Heine University Duesseldorf (study number: “2024-2739-andere Forschung erstvotierend”) and registered in the German Clinical Trials Register (DRKS00034910). The primary endpoint of this study was the measured time required to perform a MR. The secondary endpoints were the number of predefined DRPs detected and the outcomes of the questionnaire, in which participants were asked to rate statements on the use of CDSS. The complete study procedure and time schedule is illustrated in Figure 1.

### 2.2. General Procedure

The study was conducted entirely online, with all participants connected via “Cisco Webex Meetings”. A separate invitation link was provided to each participant for each study day in advance via e-mail. On each study day, the study director and four other employees from the Institute of Clinical Pharmacy and Pharmacotherapy were available to assist with any technical issues and to answer any questions arising from the participants in the chat bar. Participants in both groups were connected online at the same time on both training days (days 1 and 3 of the study). To prevent participants from identifying each other, the camera and microphone functions were deactivated in advance, and the participants were required to log in using a pseudonym. On the other two days, study days 2 and 4, the participants were required to arrange a fixed time with the study director during the recruitment phase, after which they were connected to a single virtual room. In this study, no patient interviews were conducted by participants before and after a MR. However, important patient statements about their symptoms and medications could also be found in the documents created.

### 2.3. Patient Cases

All four patient cases were fictional, designed and reviewed by the study director and four other members of the Institute (A.D., F.K., M.S., S.S., S.L.). The cases were designed to be of equal difficulty, so that, for example, the number of predefined DRPs was very similar. The cases created were multimorbid, elderly patients with different clinical profiles. The patient cases were created and discussed by the five members of the Institute without the use of “MediCheck”. All cases were only cross-checked in “MediCheck” afterwards. Patient case 1 had five chronic diseases with nine medications, patient case 2 had six chronic diseases with nine medications, patient case 3 had five chronic diseases with nine medications and patient case 4 had seven chronic diseases with eleven medications. An answer key was created for each patient case and fixed criteria were defined for the standardized allocation of a score. Once the cases were created, four external pharmacists not associated with the Institute peer-reviewed the plausibility of the cases and made recommendations. The members of the Institute checked the recommendations in detail and took them into consideration where appropriate. All patient cases are listed in Appendix A.

### 2.4. Documents from the Federal Chamber of Pharmacists (“BAK”)

During the training on the first day of the study, three documents from the BAK were presented that were relevant for conducting a MR. The first document dealt with the patient’s data collection. In this document, all problems and symptoms described by the patient, medication, diagnosed diseases, and other points that were relevant for a MR were documented. For the MR of patient cases, participants have received such a completed document in advance. The second BAK document was used to document any DRPs that were detected during the MR and considered relevant. The third BAK document was a results report for the general practitioner of the fictitious patients. Predefined DRPs that needed to be clarified with the general practitioner were recorded there and suggestions for solutions were given. The last two documents were completed by the participants for the patient cases on study days 1 and 2 and considered for the evaluation of the results.

### 2.5. CDSS “MediCheck”

“MediCheck” is a commercial, web-based medical product that can support pharmacists in conducting MRs. “MediCheck” can be accessed via any web browser, but according to the developer, it works best on “Google Chrome” and “Mozilla Firefox”. After logging in, a starting page appears on which either a new patient case can be created or previously saved cases can be edited and, if necessary, shared with colleagues (“case sharing” function). The input screen for documenting patient data in “MediCheck” is divided into 5 categories. The first category contains basic patient information, such as the patient’s first and last name, age, and gender. The second category lists all medications, both prescription and non-prescription. More detailed information such as the pack size, dosage, dosage regimen, dosage form, time of administration, and the indication for the respective medication can also be documented. In the third section symptoms reported by the patient can be documented. In category four all available laboratory values such as creatinine, cholesterol, and blood sugar levels, as well as vital signs such as blood pressure and heart rate, can be documented. Body height and weight can also be documented in this section. In the fifth category, all diagnosed diseases, allergies, and living conditions can be documented. Once all patient information has been entered, “MediCheck” can start an analysis. For the analysis, “MediCheck” refers to an extensive database containing numerous specialized information and guidelines. “MediCheck” analyses various aspects, such as drug–drug interactions, drug–disease interactions, contraindications, incorrect dosages, incorrect times of administration, etc. The analysis typically takes just a few seconds. After “MediCheck” has performed the analysis, a new page opens, which contains a summary of the patient details and a list of the DRPs. The DRPs are divided into four different risk categories, which are also highlighted in different colors. For each DRP, the cause and potential consequences of that DRP are given. Furthermore, one or more suggested solutions to eliminate the DRP are suggested. The user can decide which, if any, of the suggested DRPs are appropriate and can also change the predefined recommendations.

### 2.6. Measurement Instruments

#### 2.6.1. Time Measurement

Participants had a maximum of 90 min to solve the patient case on each day. Time measurement started with the upload of the download link containing the patient cases. Time was stopped as soon as the results, in the form of completed documents, were sent by the participants and received by the study director.

#### 2.6.2. Measurement of Predefined Relevant DRP

Participants’ performance was assessed based on the two completed BAK documents (study days 1 and 2) and the two PDF documents generated by “MediCheck” (study day 4). The study director and four other members of the Institute (A.D., F.K., M.S., S.S., S.L.) discussed the cases in advance and produced an answer key for each case to ensure that the evaluation was standardized and as objective as possible. The predefined DRPs were selected according to their clinical relevance for the individual patient cases. A maximum of two points could be allocated for each predefined relevant DRP. One point was allocated if the relevant DRP was correctly identified, and a further point was allocated if the participants provided an appropriate solution to the DRP. Patient case 1 had six DRPs and thus a maximum score of 12 points. Patient cases 2 and 3, each had five defined relevant DRPs and therefore each had a maximum score of 10 points. Patient case 4 also had five relevant DRPs. As not all cases had the same number of predefined DRPs, the evaluation was conducted on a percentage basis.

#### 2.6.3. Questionnaire

At the end of the last study day, participants were asked to complete a questionnaire. This contained three questions on demographics and nine statements to be rated. The demographic questions were related to age, gender, and work experience in a community pharmacy. The nine statements related to the implementation of a MR with and without the use of a CDSS. They were measured using a five-point Likert scale (1 = “strongly disagree”, 2 = “disagree”, 3 = “neither agree nor disagree”, 4 = “agree”, 5 = “strongly agree”). The statistical analysis of the statements was presented in a forest plot. The nine statements are shown in Table 1.

### 2.7. Procedure of Individual Days

#### 2.7.1. Study Day 1: Training Day with “BAK” Documents

First, the participants received approximately 60 min of training from an external pharmacist of “pharma4u GmbH”. The pharmacist was suitably qualified to provide training for pharmacists, as she regularly provides training courses on MRs on behalf of the Professional Chamber of Pharmacists. In addition to the general recommendations regarding MRs, the training also included guidance on how to use the BAK documents to document the results of a MR. The content of the training was coordinated in advance with the members of the Institute of Clinical Pharmacy and Pharmacotherapy. During the 60 min session, participants were permitted to submit questions via the chat function, which were then answered by the lecturer.

Following the training provided by the lecturer, the participants were instructed to perform a MR for patient case 1. The participants were provided with the patient case, which included all essential data, such as the current medication, symptoms, diagnosed diseases, laboratory and vital parameters, and other pertinent patient information. The data were made available to each participant simultaneously via a download link in the chat function of “Cisco Webex Meetings”. The time measurement began when the download link was shared with the participants. Furthermore, the download link provided access to the two BAK documents, which participants were asked to complete to document their results. Participants were permitted a maximum of 90 min to complete the MR. Except for the utilization of a CDSS, participants were permitted to employ all available resources for the purpose of the MR. Participants were informed that they should send their results to the study director by e-mail immediately after they had finished the MR. Furthermore, participants were asked to confirm in the chat function of “Cisco Webex Meetings” that they had sent the e-mail with the results to the study director in case there was a delay between sending the participant’s e-mail and receiving the e-mail by the study director. Once the two completed BAK documents had been sent to the study director, the study day was considered complete.

#### 2.7.2. Study Day 2: Medication Review Without Using “MediCheck”

As soon as the participants registered at the scheduled time, they were assigned to a virtual room by the Institute members, which had been created just for the respective participant. In this virtual room, the participants were welcomed by the study director (A.D.) and another member of the Institute (A.B.) informed them about the day’s program. To ensure a standardized and uniform welcome and briefing for each participant, a script was prepared in advance and used by the study director. The study director clearly explained to each participant that the use of a CDSS was not permitted on that day. After all organizational procedures had been clarified, participants were provided with a download link. Participants in “Group 1” received the download link for patient case 2, while participants in “Group 2” received the download link for patient case 3. The download link contained a file with all relevant information on the respective patient and the two BAK documents to be completed by the participants. Once participants confirmed that the files could be opened, the timer was started, and the participants had a maximum of 90 min to complete the MR. Throughout the entire time participants were conducting the MR, they were connected in the virtual room so that the study director or Institute staff could respond to any questions or problems as quickly as possible. As soon as the participants had finished documenting the MR, the results, which were recorded in two BAK documents, were e-mailed to the study director. In addition, the participants had to confirm again in the chat function that they had completed the case and sent the results by e-mail. When the study director or the Institute staff confirmed that the e-mail had been received, the participants were able to log out of the online meeting and the study day was completed for them.

#### 2.7.3. Study Day 3: Training Day with “MediCheck”

The procedure of the third study day followed the chronology of the first study day. Again, all participants from “Group 1” and “Group 2” were connected at the same time and received approximately 60 min of training from the same lecturer, as on the first training day. This time, however, the content of the training was on the use of the CDSS “MediCheck”. As before, the content of the training was coordinated in advance with the members of the Institute to ensure that the most important points for using the tool were mentioned to the participants. During the training, there was again the opportunity for participants to ask questions via the chat function.

This was followed by a MR for patient case 4, which all participants were asked to conduct. The conditions and requirements for conducting the MR were the same as on the first day, except that this time, the CDSS “MediCheck” could also be used. Patient case 4 was shared with all participants simultaneously in “MediCheck” by the study director via a so-called “case sharing” function. This time, the results were not documented in the BAK documents, but in “MediCheck” itself. Two portable document format (PDF) documents have been created by the software that correspond to the BAK documents when the MR has been completed. These PDF documents were then e-mailed to the study director. After a maximum of 90 min, participants who had not yet finished the MR were asked to save the status of their MR and send the results to the study director. After sending the PDF documents and the notification in the chat that the e-mail had been sent to the study director, the participants could log out and the third study day was completed. The results for patient case 4 were not analyzed. The aim of the MR for patient case 4 was to create the same setting for study day 4 as it was for study day 2 and to ensure that the participants had already carried out an analysis with “MediCheck” and were generally able to orientate themselves in “MediCheck” for study day 4.

#### 2.7.4. Study Day 4: Medication Review with “MediCheck“

The procedure was the same as on study day 2; participants were welcomed individually in a virtual room by the study director (A.D.) and a member of the Institute (A.B.) informed them about the day’s program. Participants in “Group 1” received patient case 3 using the “case sharing” function via “MediCheck” and the participants assigned to “Group 2” received patient case 2 using “case sharing” via “MediCheck”. On this day, unlike on study day 2, participants were also able to use the CDSS “MediCheck” for their MR. As on study day 3, the results were saved as PDF documents and e-mailed to the study director. The start time was the case sharing by the study director and the time was stopped as soon as the e-mail with the results was sent to the study director. Again, participants had a maximum of 90 min to solve the case and gave short feedback in the chat that the e-mail had been sent. After the documents were sent to the study director, participants were asked to complete a questionnaire. The study was completed when the participants returned the completed questionnaire by e-mail.

### 2.8. Data Protection, Analysis and Statistical Methods

Each participant was given a pseudonym before the start of the study. All data for the evaluation of the MR and the questionnaire were available in pseudonymized form. During data analysis, a coding list was used to identify which participant had which pseudonym. Only the study director (A.D.) and one other member of the Institute (A.B.) had access to the coding list. The coding list was completely and irreversibly deleted after the data had been analyzed. After the deletion of the coding list, the data were available in an anonymized form, so that it was not possible to draw conclusions about individual participants.

The randomization of the participants into “Group 1” and “Group 2” was performed using “Microsoft Excel” with the “RAND function”. “Microsoft Excel (Version 16.90)” was used for data collection and “R” programming language for data analysis. The post hoc power analysis was performed using “G*Power” software program (Version 3.1) [24,25]. With a power of 80%, an alpha of 0.05, and a medium to large effect size according to Cohen’s d of 0.75, a total number of at least 48 participants was calculated.

Statistical analyses were performed separately according to both the intention-to-treat analysis and the per-protocol analysis. For patient case 1, a two-sided Mann–Whitney test was performed between “Group 1” and “Group 2” to compare the two groups in terms of the time required to perform a MR. For patient case 2, a one-sided Mann–Whitney test was performed between “Group 1” (without CDSS) and “Group 2” (with CDSS). A one-sided Mann–Whitney test was also performed for patient case 3 (“Group 1” with CDSS, “Group 2” without CDSS). A significance level of alpha = 0.05 was considered in all statistical tests. The same statistical methods were used to compare the detected predefined DRPs between the groups.

In the questionnaire, the demographic data (age, gender, professional experience in community pharmacy) were analyzed as percentages, sorted by group, and presented in a table. The Likert scale for each of the nine statements was evaluated using the arithmetic mean and a 95% confidence interval (CI). All means and 95% CIs were presented in a forest plot. Consensus on a statement was assumed to be reached if the 95% CI did not intersect the vertical line “3” in the forest plot.

## 3. Results

### 3.1. Participant Characteristics

A total of 122 pharmacists from across Germany expressed interest in the study and requested further details. Of the 122 interested pharmacists, 71 provided written consent to participate in the study and in the privacy policy agreement. The 71 pharmacists were then randomly assigned to two groups (“Group 1” and “Group 2”). Five pharmacists withdrew before the start of the study, and four were unable to provide full data for the entire study period due to technical problems, resulting in a complete data set of sixty-two participants. However, we have included the incomplete data sets of the four participants according to the intention-to-treat analysis and excluded them in the per-protocol-analysis. Table 2 shows the demographic characteristics of “Group 1” and “Group 2”. Both groups, “Group 1” and “Group 2”, demonstrate a comparable distribution regarding gender, age, and professional experience in community pharmacy.

### 3.2. Time Required to Conduct the Medication Review

**Figure 2:**
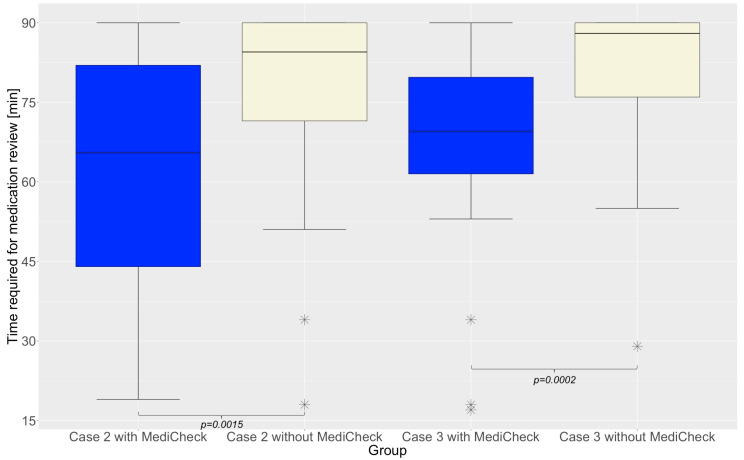
Success in quantity of performing the MR: Time required to complete the medication review for patient case 2 and 3. Horizontal line represents median. Outliers are indicated by asterisks; MR = medication review.

The results of the primary endpoint show that participants without the use of the CDSS took 30.7% more time for the MR of patient case 2 than with the use of a CDSS. For patient case 3, the participants without a CDSS needed 25.7% more time than the participants who completed this case with the CDSS “MediCheck”. The results are presented in Table 3. To check whether the two groups were equally strong and comparable with each other, patient case 1 was completed by both groups under the same conditions, and the results demonstrated that both groups were comparable as they did not differ significantly in time required (*p* = 0.2026). Regarding patient case 2, “Group 2”, which was permitted to utilize the CDSS “MediCheck”, required significantly less time for processing than “Group 1”, which resolved the case without “MediCheck” (65.5 min vs. 84.5 min; *p* = 0.0015). However, patient case 3 was reviewed by “Group 1” with “MediCheck” and by “Group 2” without “MediCheck”. Similarly, the case was completed at a significantly faster rate with the use of “MediCheck” (69.5 min vs. 88 min; *p* = 0.0002), as shown in Figure 2.

**Figure 3:**
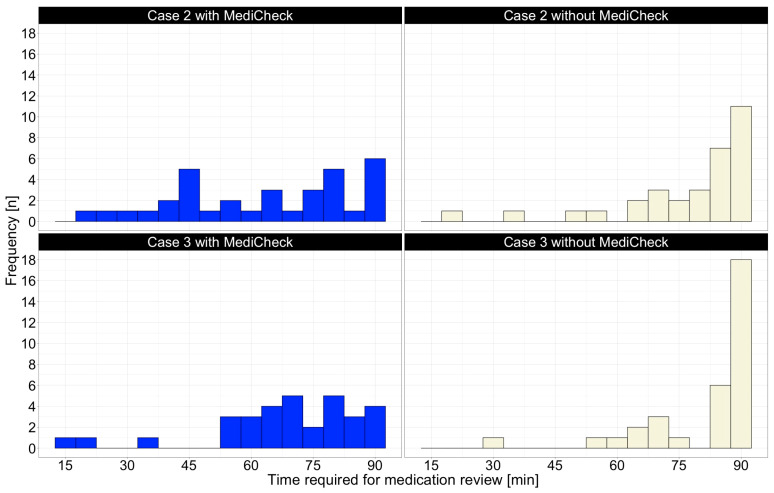
Histograms of patient cases 2 and 3 with and without CDSS for the time required to perform a MR; CDSS = clinical decision support system, MR = medication review.

For a more detailed look at the primary endpoint, Figure 3 shows histograms of the distribution of the participants’ time requirements for a MR. For patient case 2, participants without “MediCheck” were required to utilize the maximum specified time of 90 min with considerably greater frequency than the participants with “MediCheck”. With patient case 3, a difference in the distribution between “Group 1” and “Group 2” is also observable. In this case, significantly fewer participants required the maximum time of 90 min to complete the case with “MediCheck” than without “MediCheck”, as shown in Figure 3.

### 3.3. Predefined Relevant Drug-Related Problems (DRPs) Detected

**Figure 4:**
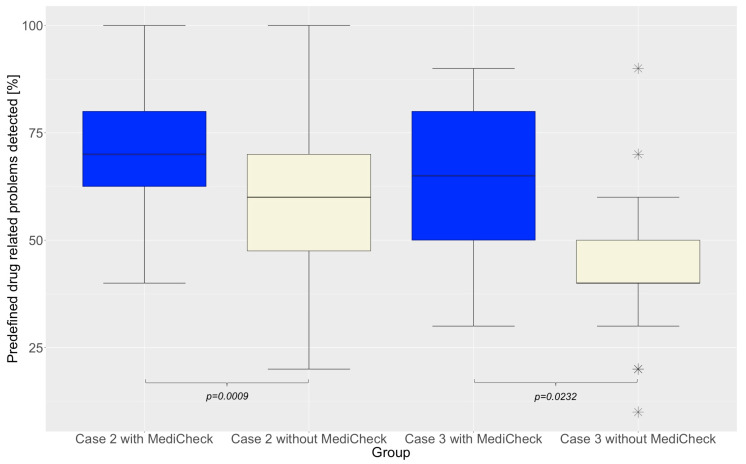
Quality success of MR: Percentage of predefined DRP numbers detected for patient case 2 and 3. Horizontal line represents the median. Outliers are indicated by asterisks. MR = medication review, DRP = drug-related problem.

**Figure 5:**
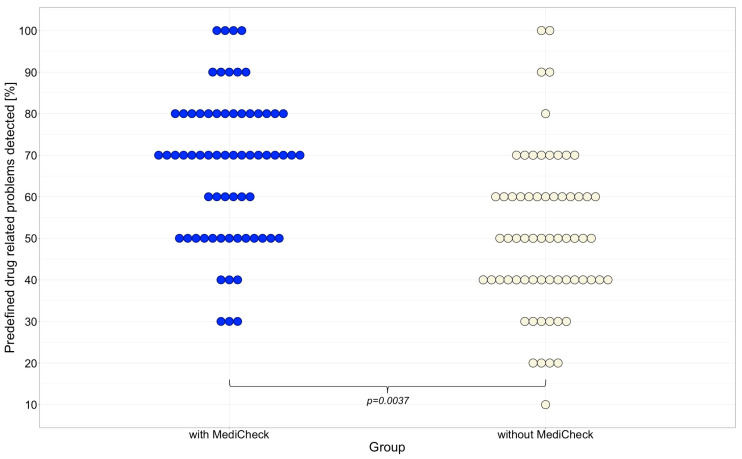
Distribution of the quality success of the MR as predefined DRPs detected by the participants with and without CDSS for both cases 2 and 3. Each dot represents one participant; MR = medication review, DRPs = drug-related problems, CDSS = clinical decision support system.

The secondary endpoint was defined as the number of predefined DRPs detected. Significantly more predefined DRP were identified with the use of the CDSS “MediCheck” than without (70% vs. 50%; *p* = 0.0037). There is no significant difference between “Group 1” (50%) and “Group 2” (50%) in the first patient case in terms of DRPs (*p* = 0.3615). Both groups are, therefore, also comparable in terms of performance. “Group 2” was demonstrably more effective in detecting predefined DRPs in patient case 2 when using “MediCheck” than “Group 1” without using “MediCheck” (70% vs. 60%; *p* = 0.0009). In patient case 3, the use of “MediCheck” led to a significant increase in the detection of predefined DRPs (*p* = 0.0232), with 65% detected with “MediCheck” compared to 40% without, as shown in Figure 4. Table 4 lists the DRP scores obtained for each patient case. Figure 5 also shows that the distribution of the detected predefined DRPs shifts towards 100% when a CDSS is used. This indicates a significant improvement in the quality of the MR.

### 3.4. Results of the Questionnaire

Another secondary endpoint was defined as the participants’ rating of the statements in the questionnaire. For all nine statements, there was a clear consensus among the participants who generally welcomed the use of the CDSS in MRs.

**Figure 6:**
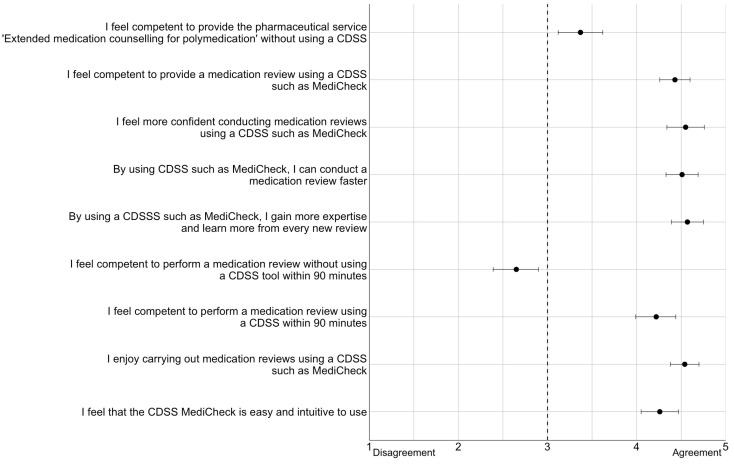
Display of the results of the questionnaire in a forest plot. The black dot represents the arithmetic mean. The horizontal lines to the left and right of the box represent the 95% CI. Consensus on a statement is reached when the 95% CI does not intersect the vertical line at 3 on of the x-axis of the forest plot. Consensus was reached on all statements; CI = confidence interval.

The questionnaire was analyzed using a forest plot, as shown in Figure 6. A consensus was found for all statements. The arithmetic means of the statements 2, 3, 4, 5, 7, 8, and 9 are evenly located between “agree” and “strongly agree”, indicating a very clear position on the statement. There is a consensus on the statement that a MR within 90 min does not seem possible without the use of a CDSS such as “MediCheck”. However, by using “MediCheck”, it is well possible that the MR will take place within 90 min. In addition, the participants felt, on average, less competent to carry out a MR without using a CDSS (Statement 1) than with a CDSS (Statement 2).

## 4. Discussion

In this randomized controlled study, we found that pharmacists who used a CDSS such as “MediCheck” performed a MR faster than those who did not use a CDSS. Participants without a CDSS took 30.7% longer to complete a MR for patient case 2 and 25.7% longer to complete patient case 3 than those using a CDSS. Furthermore, the MRs in which a CDSS was used revealed more predefined DRPs than the MRs without CDSS. In patient case 2, 16.7% more predefined DRPs were detected with CDSS use than without, and in patient case 3, 62.5% more predefined DRPs were detected. Moreover, the participating pharmacists had a clearly positive opinion of CDSSs and their use for performing MRs. Not only do pharmacists feel more confident performing a MR with a CDSS than without, but they also feel that the MR can be performed faster with a CDSS.

To the best of our knowledge, this is the first study to investigate the impact of a CDSS on MRs in community pharmacies with a focus on time. In their pilot study, Riley et al. [26] investigated the impact of a CDSS on pharmacists’ work in community pharmacies and mentioned in an aside that no differences were observed in the time taken by pharmacists to perform MRs. We were able to disprove this statement in our study, which included a larger number of community pharmacists who received the same standardized cases.

The study also showed that the quality of the MRs increased with a CDSS and that predefined DRPs were more likely to be detected. In patient case 2, participants without a CDSS detected 60% of the predefined DRPs, whereas participants with a CDSS detected 70%. This is a relative difference of 16.7%. For patient case 3, there is even a relative difference in the detection of predefined DRP of almost 62.5%. This is in accordance with the results of previous studies using CDSSs in community pharmacies. The pilot study by Riley et al. and the pre-to-post analysis by Verdoorn et al. have also shown that more DRPs are detected in performing a MR using a CDSS than without such a tool [26,27].

The increase in efficiency and effectiveness of the MRs as a result of the CDSS is also consistent with the subjective impressions of the participants, which can be observed from the results of the questionnaire. In principle, there was a consensus among the participants that they felt competent to perform a MR even without using a CDSS. However, the participants also stated that they felt more confident performing a MR when supported by a CDSS. Most participants did not feel able to perform a MR within 90 min without a CDSS, whereas they felt able to perform a MR within 90 min with a CDSS. Overall, the use of a CDSS for MRs was well received by the participating pharmacists. Surveys on CDSSs have already been conducted in Malaysia and Australia, for example, where pharmacists were similarly positive about CDSSs and welcomed the establishment of such digital tools for the performance of MRs [28,29].

Although this is a prospective, randomized controlled study, it has limitations. First, the number of participants should be considered, which does not appear to be particularly high with a total of 66 participants. The reason for the limited number of participants is the time required for the study, which is why only 71 (58.2%) of the 122 pharmacists who were initially interested took part and the data from 66 participants (54.1%) could be analyzed. Therefore, a post hoc power analysis was performed using the determined medians, standard deviations, and sample sizes, in which a power of over 95% was calculated for each of patient cases 2 and 3. Since the usual target value for power should be between 80 and 90%, the sample size appears to be large enough. However, we recommend a larger number of participants for future studies to corroborate the results. Secondly, the participants in the study are not representative of the overall picture of pharmacists in Germany. Most pharmacists were informed about the participation in the study via the newsletter of the company “pharma4u GmbH”, so they are already pharmacists who had a greater interest in conducting MRs. However, in our study we explicitly looked for pharmacists who were neither familiar with the software nor with the routine performance of MRs and took this into consideration in the inclusion criteria. One argument in favor of the representativeness of the study is that pharmacists from all over Germany took part, from both urban and rural areas. Thirdly, the CDSS “MediCheck” displays many DRPs, including some that are not of major clinical importance. This also increases the likelihood that the predefined DRPs will be recognized by the system, because, in principle, almost all potential DRPs are displayed. However, the evaluation of the secondary endpoint of the predefined DRP showed that the pharmacists were able to identify relevant DRPs from less relevant DRPs and to indicate the predefined, more relevant DRPs in the MR.

## 5. Conclusions

In conclusion, the results of the study show that the additional use of a CDSS such as “MediCheck” is a useful support for performing a MR. On the one hand, pharmacists are open to new tools that support their work and make processes more efficient, and on the other hand, patients benefit from the software, which can further improve the quality of MRs. Given the demographic trends in Germany, it is expected that the number of polymedicated patients will increase in the future, as will the need for medication reviews to ensure the safety of the population’s drug therapy. It is therefore essential to optimize processes and services such as MRs.

## Figures and Tables

**Table 1:** The nine statements of the participants’ questionnaire.

Statement 1	I feel competent to provide the pharmaceutical service “Extended medication counseling for polymedication” without using a CDSS.
Statement 2	I feel competent to provide a medication review using a CDSS such as “MediCheck”.
Statement 3	I feel more confident conducting medication reviews using a CDSS such as “MediCheck”.
Statement 4	By using a CDSS such as “MediCheck”, I can conduct a medication review faster.
Statement 5	By using a CDSS such as “MediCheck”, I gain more expertise and learn more from every new review.
Statement 6	I feel competent to perform a medication review without using a CDSS tool within 90 min.
Statement 7	I feel competent to perform a medication review using a CDSS within 90 min.
Statement 8	I enjoy carrying out medication reviews using a CDSS such as “MediCheck”.
Statement 9	I feel that the CDSS “MediCheck” is easy and intuitive to use.

CDSS = clinical decision support system.

**Table 2:** Demographic characteristics of study participants.

	Group 1 (*n* = 32) *n* (%)	Group 2 (*n* = 34) *n* (%)
Gender		
Female	27 (84.4)	26 (76.5)
Male	5 (15.6)	8 (23.5)
Age range		
Under 30 years	5 (15.6)	6 (17.6)
30–40 years	8 (25.0)	8 (23.5)
40–50 years	10 (31.3)	6 (17.6)
Over 50 years	9 (28.1)	14 (41.2)
Years of Professional experience in community pharmacy		
Less than 2 years	5 (15.6)	4 (11.8)
2–5 years	2 (6.3)	4 (11.8)
5–10 years	6 (18.8)	5 (14.7)
More than 10 years	19 (59.4)	21 (61.8)

**Table 3:** Time required to conduct the medication review with and without “MediCheck” for each patient case.

	Group 1 Minutes	Group 2 Minutes	*p*-Value
Patient case 1			
“MediCheck“ used?	no	no	
Median (MAD)			
Intention-to-treat analysis	64.5 (19.3)	73.0 (16.3)	0.2026
Per-protocol analysis	64.5 (19.3)	73.5 (16.3)	0.1464
Patient case 2			
“MediCheck“ used?	no	yes	
Median (MAD)			
Intention-to-treat analysis	84.5 (8.2)	65.5 (30.4)	0.0015
Per-protocol analysis	84.0 (8.9)	65.5 (31.9)	0.0048
Patient case 3			
“MediCheck“ used?	yes	no	
Median (MAD)			
Intention-to-treat analysis	69.5 (14.1)	88.0 (3.0)	0.0002
Per-protocol analysis	69.5 (15.6)	88.0 (3.0)	0.0003

MAD = median absolute deviation.

**Table 4:** Detected predefined DRPs with and without “MediCheck” as a CDSS for each patient case.

	Group 1 Predefined DRP Detected (%)	Group 2 Predefined DRP Detected (%)	*p*-Value
Patient case 1			
“MediCheck“ used?	no	no	
Median (MAD)			
Intention-to-treat analysis	50 (19)	50 (12)	0.3615
Per-protocol analysis	50 (19)	50 (12)	0.4846
Patient case 2			
“MediCheck“ used?	no	yes	
Median (MAD)			
Intention-to-treat analysis	60 (15)	70 (15)	0.0009
Per-protocol analysis	60 (15)	70 (15)	0.0001
Patient case 3			
“MediCheck“ used?	yes	no	
Median (MAD)			
Intention-to-treat analysis	65 (22)	40 (15)	0.0232
Per-protocol analysis	70 (15)	40 (15)	0.0267

MAD = median absolute deviation, DRP = drug-related problem.

## Data Availability

The data set presented in this study is available from the corresponding author upon reasonable request.
